# Cost-Effectiveness of Corneal Collagen Crosslinking for Progressive Keratoconus: A Brazilian Unified Health System Perspective

**DOI:** 10.3390/ijerph21121569

**Published:** 2024-11-26

**Authors:** Lucca Ortolan Hansen, Renato Garcia, André Augusto Miranda Torricelli, Samir Jacob Bechara

**Affiliations:** Division of Ophthalmology, School of Medicine, University of São Paulo, São Paulo 01246-903, SP, Brazil

**Keywords:** keratoconus, cost-effectiveness, incremental cost-effectiveness ratio, incremental net monetary benefit, keratoconus, corneal crosslinking, quality-adjusted life years, SUS, Brazilian Unified Health System

## Abstract

Keratoconus is a burden to health systems and patients worldwide. Corneal collagen crosslinking (CXL) treatment has been shown abroad to be cost-effective for treating progressive keratoconus. However, no cost-effectiveness studies have been performed in Brazil. The aim of this study was to assess the cost-effectiveness of corneal collagen crosslinking (CXL) compared with the conventional treatment for progressive keratoconus from the Brazilian Unified Health System (SUS) payer’s perspective. A lifetime microsimulation model was utilized to compare the lifetime costs and quality-adjusted life years for patients undergoing corneal collagen CXL or conventional treatment. Two groups of 5000 18-year-old patients were simulated, with one group receiving corneal CXL at the outset and the control group remaining untreated. The TreeAge Pro Healthcare 2024 software was used for modeling and analysis. Corneal collagen CXL demonstrated superior cost-effectiveness compared to the conventional treatment, with an incremental cost-effectiveness ratio of 58.26 USD/quality-adjusted life years (QALY) gained (95% CI: 58.17–58.36) and a positive incremental net monetary benefit of USD 11,613.82 (95% CI: 11,605.66–11,621.99). CXL significantly reduced the number of required corneal transplants, with a mean of 968.8 (95% CI: 959–978.58) fewer transplants per 10,000 eyes treated. The variable with the most significant impact on the incremental net monetary benefit was the duration of the CXL effect. This study concluded that corneal CXL is a highly cost-effective intervention for progressive keratoconus within the Brazilian SUS. These findings advocate for broader accessibility to this vision-saving treatment within the SUS.

## 1. Introduction

Keratoconus (KC) is a bilateral, ectatic corneal disease characterized by corneal steepening and thinning [1,2]. These progressive changes induce myopia and irregular astigmatism, ultimately impairing vision and diminishing patients’ quality of life [2,3]. This disease can lead to difficulties in education, employment, productivity, and overall well-being [3]. In severe cases, corneal scarring can exacerbate vision impairment [1,2,4,5,6,7].

### 1.1. Background

Historically, KC treatment has been expensive, often necessitating lifelong visual rehabilitation with glasses, contact lenses, and surgery. Corneal transplants are also costly and frequently require additional procedures, tests, and consultations. Researchers have investigated these costs to better understand the socioeconomic burden of this disease on healthcare systems [8,9,10,11].

Emerging evidence suggests that implementing a crosslinking (CXL) treatment in the Netherlands is correlated with a subsequent reduction in corneal transplants among KC patients [12].

### 1.2. Collagen CXL Treatment

CXL is an effective, safe, and reproducible treatment modality for progressive KC in both pediatric and adult populations, demonstrating long-term efficacy [13,14,15,16,17]. Preventing further disease progression is theoretically ideal, as it potentially reduces the need for expensive corneal transplants and visual rehabilitation. This preventive approach could also minimize the frequency of ophthalmologic visits and help patients maintain better work productivity.

### 1.3. Early Detection and Diagnosis

Early-stage KC can be challenging to diagnose [18,19]. However, advanced technologies, such as corneal tomography, enable posterior corneal evaluations and the detection of subtle changes in the epithelium, allowing for early disease detection [2,20,21,22,23,24] (8–11). Such technologies could increase diagnostic sensitivity and specificity, allowing ophthalmologists to monitor at-risk patients more effectively [22].

### 1.4. Treatment Considerations

The literature debates what constitutes KC progression [2,25] and whether treatment should be limited to those with progression [2,26,27]. However, most experts agree that progressive corneal changes in KC warrant a CXL treatment across all age groups [2,14].

### 1.5. Cost-Effectiveness Studies

Cost-effectiveness studies are valuable tools for comparing treatments and understanding the economic implications of public health decisions, such as the adoption of CXL treatments [28]. International studies have demonstrated the cost-effectiveness of CXL compared with observation alone in cases of progressive KC.

### 1.6. Current Situation in Brazil

Few studies on the epidemiology of KC in Brazil exist [29,30], and KC’s incidence and prevalence are mostly unknown, considering the most cited prevalence statistic (54:100,000) from the USA [31]. Using the prevalence figures from Hofstetter, it is conservatively estimated that there are 110,400 patients with KC in Brazil and 4000 new cases per year [30,32].

Despite the approval of the Brazilian Unified Health System (SUS) National Committee for Technology Incorporation (CONITEC) for CXL treatment [33], cost-effectiveness studies utilizing local public health cost data for progressive KC are lacking.

## 2. Materials and Methods

### 2.1. Modeling Approach

Two identical groups of 5000 simulated 18-year-old patients (10,000 eyes per group) with progressive KC were created. One group received CXL at the beginning of the simulation, whereas the control group (conventional group) remained untreated.

The conventional treatment of KC in Brazil’s public system is restricted to follow-up, pruritus management, crosslinking, or corneal transplants. Intrastromal corneal ring segments, epithelium-on CXL (CXL with no removal of the epithelium), and “CXL-plus” (CXL with simultaneous PRK treatment) have not been approved yet for SUS use. The SUS does not provide keratoconus ophthalmic lenses (spectacle, rigid gas-permeable, scleral). Therefore, our model simulated conventional treatment according to the local reality from the SUS payer’s perspective and did not account for intracorneal ring segments, lenses, crosslinking-plus, antihistamine eye drops, preservative-free eye drops, and other non-SUS approved treatment costs.

The keratoconus modeling, progression, and clinical characteristics of the groups were based on two randomized controlled trials of CXL for progressive KC [15,34,35] performed in a similar cost-effectiveness analysis study by Godefrooij et al. [9]. The base model assumed that CXL’s effect would last ten years, and an effectiveness rate of 99% was utilized. Probabilistic modeling was performed using TreeAge Pro Healthcare 2022 software.

### 2.2. Health State Simulation

During the simulation, patients transitioned between health states according to probabilities derived from the RCTs and the patient’s visual acuity category. The transition probabilities, best-corrected visual acuity groups, and transplant failure rates used were those of Godefrooij’s published model [9].

The simulations were run until the cohort reached 90 years of age. The number of stages was determined based on the availability of age-specific mortality probabilities from the 2022 mortality table published by the Brazilian Institute of Geography and Statistics [36]. The probability of death for a KC patient was assumed to be equivalent to that of the general population.

The patient’s risk of corneal transplantation was based on data from Godefrooij’s study and was originally from the Collaborative Longitudinal Evaluation of Keratoconus study [9,37,38]. The lack of upper-age restrictions for corneal transplants was consistent with the SUS guidelines. To the best of the authors’ knowledge, no studies comparable in nature and scope to the Australian Graft Registry report [39] exist regarding the chance of transplant failure in KC patients in Brazil. Therefore, the Australian data were used as in Godefrooij’s modeling.

### 2.3. Costs and Utilities

Utilities were accrued according to the VA (visual acuity) of the better eye, as proposed by Godefrooij et al. [9,40]. The cost information was extracted from the national health cost tables provided by the SUS [41] and summarized in Table 1 and Table 2. The costs were calculated in Brazilian reais and converted to USD via a trailing 12-month average of daily closing exchange rates (4.99, rounded to 5.00 USD/BRL); the Brazilian Central Bank website was accessed on 7 July 2024 [42].

### 2.4. Cost-Effectiveness and Sensitivity Analysis

The cost-effectiveness analysis used 5000 trials (first order) of the Monte Carlo microsimulation. It employed a public health system payer’s perspective, considering only health system costs (consultations, surgeries, examinations). A global discount rate of 5% was applied to costs and quality-adjusted life years (QALYs), following SUS’s guidelines detailed in the Economic Guidelines for Health Evaluation by the Brazilian Ministry of Health [43].

A probabilistic sensitivity analysis was conducted through microsimulation with 1000 samples (second order) to analyze possible variations in the studied variables. A tornado diagram visually represented the variable changes in relation to the incremental net monetary benefit (INMB).

The Brazilian Ministry of Health recommends a willingness-to-pay (WTP) of one to three times the per capita GDP [43]. According to the Brazilian Institute of Geography and Statistics [44], the 2021 GDP per capita was USD 8449.50 (BRL 42,247.52).

Therefore, the WTP range for the SUS should be between USD 8449.50 (BRL 42,247.52) and USD 25,348.51 (BRL 126,742.56). This study used the lowest (most conservative) WTP.

This study adhered to the tenets of the Declaration of Helsinki and received approval from the local ethics committee.

Table 1 presents the considered baseline scenario costs (minimum costs/most conservative scenario), while Table 2 outlines the additional costs considered in the tornado plot analysis.

Table 2 summarizes the complications of penetrating transplants, and their associated costs based on the SUS reference table of September 2020 and the Brazilian Ministry of Health’s Transplant Manual [41]. The complication costs were considered in the tornado plot analysis.

## 3. Results

### 3.1. Cost-Effectiveness

**Figure 1:**
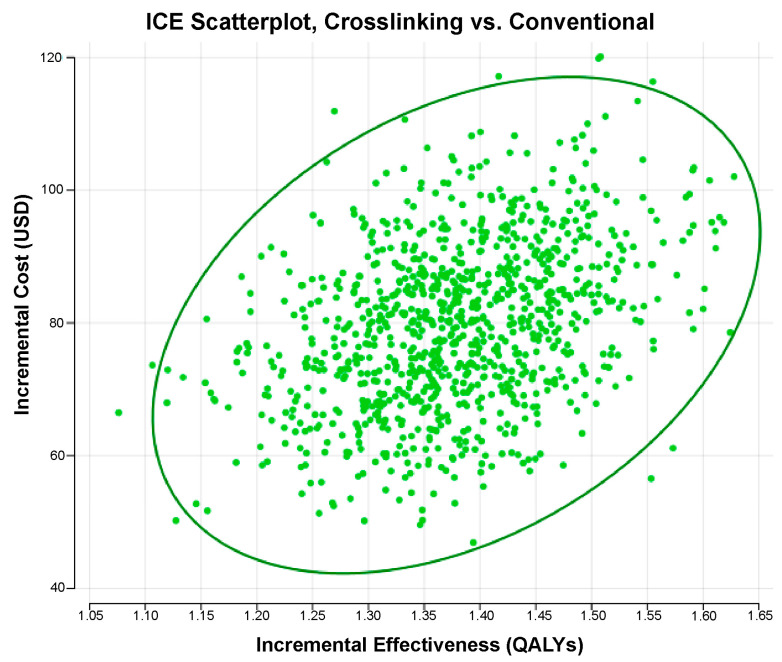
Incremental cost-effectiveness (ICE) scatterplot, crosslinking (CXL) vs. conventional. Figure 1 shows an incremental cost-effectiveness scatterplot comparing the cost-effectiveness of the CXL and conventional methods in the different simulations. The graph’s adopted confidence ellipse (darker green) was 95%. The green dots represent the simulations in which the CXL strategy was most cost-effective. The adopted willingness-to-pay cutoff was USD 8449.50 (BRL 42,247.52).

**Figure 2:**
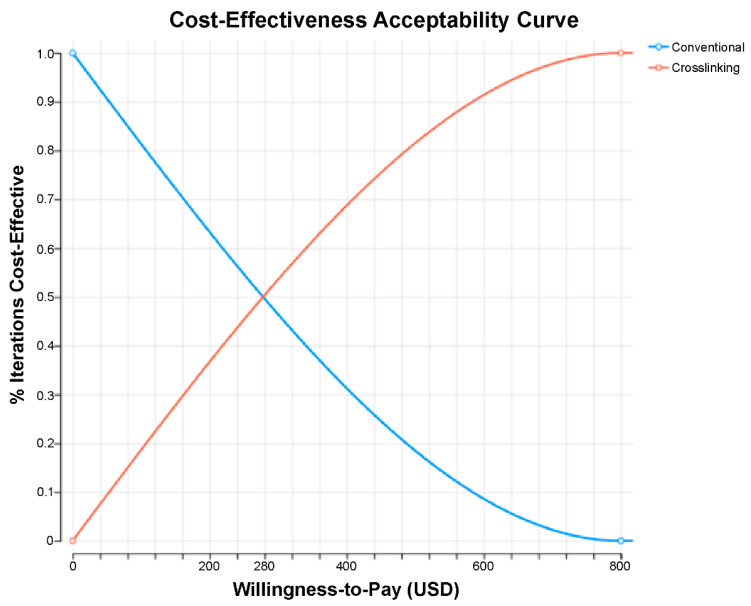
Cost-effectiveness acceptability curve demonstrated that crosslinking (CXL) was the most cost-effective strategy, with a willingness-to-pay higher than USD 800 (BRL 4000), less than one-tenth of the national gross domestic product per capita.

The cost-effectiveness analysis revealed that the CXL treatment was more cost-effective than the conventional treatment for progressive KC from the SUS payer’s perspective. An incremental cost-effectiveness scatterplot analysis (Figure 1) and a conservative willingness-to-pay cutoff revealed that the confidence ellipse fell significantly below the WTP cutoff. The cost-effectiveness acceptability curve (Figure 2) further demonstrated that the CXL treatment was the most cost-effective strategy in all the simulations above a WTP cutoff of USD 800 (BRL 4000.00).

The calculated mean INMB was USD 11,613.82 (95% CI: USD 11,605.66–USD 11,621.99) (BRL 58,069.13; 95% CI: BRL 58,028.32–BRL 58,109.95). This positive INMB, with no overlap in the net monetary benefit (NMB) confidence intervals (Table 3), demonstrates that the CXL intervention is more cost-effective than the conventional treatment (untreated cohort). The mean incremental cost-effectiveness ratio (ICER) was USD 58.26/QALY gained (95% CI: USD 58.17–USD 58.36) (BRL 291.31/QALY gained; 95% CI: BRL 290.83–BRL 291.81).

The analysis revealed a statistically significant difference among the NMB, cost, and efficacy outcomes of the conventional and CXL groups, with no overlap in their 95% confidence intervals.

### 3.2. Reduction in Corneal Transplants

Compared with the untreated group, the CXL treatment group had significantly fewer total transplants (*p* < 0.05); on average, 968.80 (95% CI: 959–978.58) transplants were avoided per 10,000 eyes treated with CXL. The CXL group had 7529.55 [95% CI: 7522.75–7536.35] total transplants vs. 8498.35 [95% CI: 8491.30–8505.40] for the conventional group. In the CXL group, first-time and bilateral transplants were also significantly less frequent (*p* < 0.05).

### 3.3. Visual Acuity Loss

At the start of the simulation, 79.9% of patients in both cohorts had good vision in at least one eye. Ten years into the simulation, 84.1% of patients in the CXL cohort still had good visual acuity in the better eye, whereas 72.8% of patients in the untreated cohort had good visual acuity in the better eye.

### 3.4. Quality-Adjusted Life Years and Costs

The costs were higher (*p* < 0.05) in the CXL group than in the untreated group (Table 3). In addition, the number of QALYs was greater (*p* < 0.05) in the CXL group than in the untreated group.

### 3.5. Probabilistic Sensitivity Analysis

**Figure 3:**
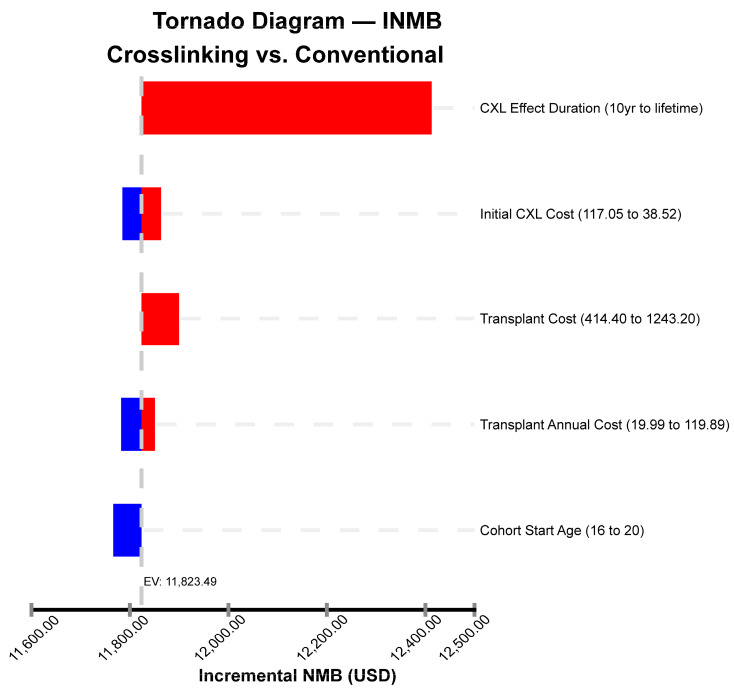
Tornado diagram—incremental net monetary benefit (INMB) of a univariable sensitivity analysis, crosslinking (CXL) vs. conventional. This graph represents the impact of the variables on the INMB. The bars are colored according to the INMB effect of each variable; red and blue show an increase and a decrease in the INMB, respectively. The adopted willingness-to-pay cutoff was USD 8449.50 (BRL 42,247.52).

The tornado plot (Figure 3) revealed that the variable with the most significant impact on the INMB was the duration of the CXL effect.

## 4. Discussion

Our cost-effectiveness analysis demonstrated that corneal collagen CXL is a highly cost-effective intervention for treating progressive KC within the SUS. We report an ICER of USD 58.26/QALY compared with the conventional treatment. This finding aligns with a growing body of evidence supporting the economic benefits of CXL across various healthcare settings.

Salmon et al. [11] conducted a cost-effectiveness analysis of CXL for progressive KC in the UK National Health Service via a 25-year model. Their base-case analysis yielded an ICER of USD 4885.10/QALY gained (GBP 3174/QALY gained, historical conversion rate for USD/GBP of 1.5391 from 28 August 2015), which was well below the commonly accepted cost-effectiveness threshold in the UK. Similarly, Leung et al. [10] reported that CXL is cost-effective in the Canadian context, with an ICER of USD 6729.84/QALY gained (CAD 9090/QALY gained, CAD/USD = 1.3507 CAD from 9 March 2017), compared with conventional management with penetrating keratoplasty.

Godefrooij et al. [9] also evaluated the lifetime cost-effectiveness of CXL for progressive KC, reporting an ICER of USD 59,822/QALY gained (EUR 54,384/QALY gained). While this ICER is higher than those reported by Salmon et al. and Leung et al., it remains within the acceptable range for cost-effective interventions in many healthcare systems. Godefrooij et al. further highlighted that the ICER decreased to USD 11,163/QALY gained (EUR 10,149/QALY gained) when a lifelong stabilizing effect of CXL was assumed, underscoring the potential for long-term cost savings.

Similarly, Lindstrom et al. [45] demonstrated the long-term economic benefits of CXL. Their lifetime economic model, which compared CXL with conventional management of KC, revealed that CXL dominated conventional treatment, reducing costs by USD 8677 per patient treated. This finding further supports the notion that CXL is a cost-effective intervention over a patient’s lifetime. Their model included lenses, glasses, and lost productivity.

It is essential to acknowledge that cost-effectiveness analyses are inherently sensitive to the model structure, discount rates, costs, underlying assumptions, and data inputs. Despite variations in modeling approaches and healthcare settings, the consistent conclusion across these studies underscores the robustness of CXL’s cost-effectiveness as a treatment strategy for progressive KC.

While previous studies have demonstrated the cost-effectiveness of CXL in other healthcare settings [9,46], this study provides the first comprehensive analysis of its economic impact within the SUS, considering this context’s unique structure, costs, and challenges.

Compared with most other studies [9,10,11,45], the more cost-effective ICER of our analysis may be attributed to the lower ratio of costs between CXL and corneal transplants in Brazil (1:5.27) compared to that abroad [9,10,11], except for in the Lindstrom et al. model, which had a similar ratio (1:5) [45].

The public health system in Brazil, known as SUS, covers most transplant procedures in the country. In 2014, the SUS spent USD 2,738,869 on 4234 keratoplasties for KC. This cost information does not consider complications or indirect costs associated with corneal transplants. Furthermore, up to 20% of all the transplants in Brazil are performed due to KC. Transplant information released by organizations in Brazil needs to be improved; therefore, the national literature is scarce [39].

CXL is still underutilized in Brazil. A recent (*n* = 267) study [29] revealed that only 8.24% of patients had been treated with CXL at presentation (mean age: 23 years), even though most patients had moderate (29.21%) or severe (58.80%) disease.

Beyond the ICER, our analysis revealed a statistically significant mean INMB of 11,613.82, favoring CXL over the conventional treatment. This positive INMB reinforces the economic attractiveness of CXL for the Brazilian SUS, indicating that the monetary value of health benefits outweighs the implementation costs. While not all the articles reported INMB findings, the positive INMB observed in our study and the favorable ICERs reported in the literature provide compelling arguments for policymakers to consider the broader adoption of CXL.

### Limitations

This study has several limitations. First, we relied on data from international studies from developed countries due to the limited availability of Brazilian data on KC clinical features, progression, transplant rates, and transplant failure. This approach might underestimate the transplant failure rate, complication rates, and the costs associated with corneal transplants from the SUS’s payer perspective.

Second, our model did not capture indirect costs (e.g., productivity losses), patient-borne expenses (e.g., transportation), or medication costs, which could underestimate the total economic burden of KC and the full benefits of CXL.

Third, due to regional differences and locally available treatment options, our model results may not apply to other populations or even Brazil’s private health system (which has different procedure costs and treatment options).

Fourth, if the SUS eventually incorporates intracorneal ring segment implantation or epithelium-on or crosslinking-plus treatments [46], a new, updated cost-effectiveness model needs to be developed to account for these treatments’ costs.

## 5. Conclusions

In conclusion, this study provides compelling evidence that corneal CXL is a highly cost-effective intervention for treating progressive KC within the Brazilian SUS healthcare system. The significant cost-effectiveness that has been demonstrated, coupled with improved visual acuity and quality of life, strongly advocates for the broader adoption of CXL as a standard of care for eligible patients. By investing in CXL, the Brazilian healthcare system can reduce the long-term economic and medical burden while improving patient outcomes and ensuring equitable access to sight-saving treatment.

## Figures and Tables

**Table 1:** Baseline scenario costs.

Procedure	Base Scenario Estimated Cost (in USD)	Cost Components
Routine cornea KC annual consultation	19.98	Tonometry; topography; cornea consultation; fundus examination.
CXL treatment	78.52	Radiation for CXL; cornea consultation.
Penetrating corneal transplant	414	Corneal transplant.
Corneal transplant follow-up	59.94	Tonometry; topography; cornea consultations; fundus examination.

Note: All costs are in USD. The baseline scenario assumes that the annual routine cost triples for the transplanted patient. Source: Costs are based on the Brazilian Unified Health System reference table of September 2020 and the Brazilian Ministry of Health’s Transplant Manual [41]. KC, keratoconus; CXL, crosslinking.

**Table 2:** Complications of penetrating transplants and costs.

Complication (Probability)	Estimated Cost	Cost Components
Acute glaucoma (4.30%)	248	Trabeculectomy; glaucoma consultations.
Suture dehiscence (4.60%)	54.40	Resuture; tonometry; cornea consultation; fundus exam.
Retinal detachment (1%)	289.00–730.90	Scleral introflexion; posterior vitrectomy; retinal mapping; tonometry; retina consultations.
Cataract (2.40%)	177.41	Phacoemulsification; cataract consultation.
Endophthalmitis (0.38%)	39.55–730.90	Intravitreal injection; posterior vitrectomy; retinal mapping; tonometry; retina consultations.
Expulsive bleeding (0.40%)	140.59	Evisceration; cornea consultations.

Note: All costs are in USD. This table was modified from the probability of complications table published by Leung et al. [10] with national costs. Source: Costs were based on the Brazilian Unified Health System reference table of September 2020 and the transplant manual of the Brazilian Ministry of Health [41].

**Table 3:** Results (per capita) of the Monte Carlo microsimulation of the comparative study (CXL vs. conventional).

Statistics	Cost (CVT)	Cost (CXL)	Effectiveness (CVT)	Effectiveness (CXL)	NMB (CVT)	NBM (CXL)
Mean	697.04	777.68	14.81	16.19	124,418.91	136,032.73
SD	11.20	14.14	0.10	0.07	851.78	557.29
Minimum	660.27	727.75	14.47	15.92	121,530.01	133,732.89
Median	696.97	777.58	14.81	16.19	124,421.46	136,041.52
Maximum	719.01	805.44	15.00	16.31	126,069.35	137.071.86
Lower Bound 95%CI	696.73	777.29	14.80	16.19	124,395.30	136,017.29
Upper Bound 95%CI	697.35	778.08	14.81	16.19	124,442.52	136.048.19

Note: All costs and NMBs in the table are listed in USD. Effectiveness is in QALYs. SD = standard deviation; CVT = conventional group; CXL = crosslinking group; NMB = net monetary benefit. A total of 1000 simulations were performed. The results were rounded to two decimals. NMB was calculated using a willingness-to-pay of USD 8449.50 (equal to the national per capita GDP).

## Data Availability

The data are contained within the article. Additional datasets are available from the authors upon request.
